# Ornidazole-Induced Recurrent Encephalopathy in a Chinese Man: A Rare Case Report and Literature Review

**DOI:** 10.3389/fneur.2021.706094

**Published:** 2021-09-09

**Authors:** Rong Tang, Jia Liang, Yuanfang Li, Tingting Wu, Yuhao Zhang, Yu Ma, Xu Liu

**Affiliations:** ^1^Department of Neurology, Zhongshan Hospital, Fudan University (Xiamen Branch), Xiamen, China; ^2^Department of Neurology, Zhongshan Hospital, Fudan University, Shanghai, China

**Keywords:** ornidazole-induced encephalopathy, ataxia, dentate nucleus, MRI, toxicity

## Abstract

Ornidazole-induced encephalopathy (OIE) is seldom seen in the clinic. In this study, we report a new case of a patient who had taken 1,000 mg ornidazole daily for nearly 4 years because of suspected diarrhea and proctitis and presented with subacute symptoms such as unsteady gait, slurred speech, and psychiatric disorder. These symptoms were significantly relieved 3 days after the patient stopped taking ornidazole. When he took this medicine again, however, similar symptoms occurred 4 months later, which were again reduced after 4 days of drug discontinuation. After the second onset, abnormal signals were identified around the aqueduct of the midbrain, around the fourth ventricle, and in the dentate nuclei of the cerebellum bilaterally. After 9 days of drug discontinuation, lesions disappeared in the magnetic resonance imaging (MRI) results. According to the clinical manifestations, imaging features, and the reduced symptoms after drug withdrawal, we clinically diagnosed the patient with OIE. This paper also reviews the literature on OIE. Only five cases (including our case) have been reported, all of whom presented with cerebellar ataxia and dysarthria and three with additional mental symptoms such as agitation and irritability. All five patients had abnormal lesions in the dentate nucleus of the cerebellum bilaterally, among whom four also had lesions in the corpus callosum and three around the periaqueduct of the midbrain. After withdrawal of ornidazole, the symptoms in all patients vanished or were alleviated, and three of them showed reduced or disappeared lesions in a head MRI reexamination. Overall, OIE has rarely been reported. Our case report and literature review show that the lesions in the cerebellum, corpus callosum, and brainstem can be reversed. The main manifestations of the lesions—cerebellar ataxia, dysarthria, and mental symptoms—quickly weaken or disappear after drug withdrawal, with good prognosis. Nevertheless, clear pathogenesis has yet to be further investigated.

## Introduction

Nitroimidazole drugs, mainly including metronidazole, tinidazole, and ornidazole, with anti-anaerobic and antiprotozoal effects, are generally used for peptic ulcers. The common adverse reactions of nitroimidazoles include gastrointestinal symptoms such as nausea, vomiting, metallic taste, and abdominal discomfort. The adverse reactions of the nervous system are mostly manifested as peripheral nerve damage, especially sensory nerve damage (1). Metronidazole-induced encephalopathy (MIE) is a serious adverse reaction of the central nervous system that is mainly manifested as ataxia, dysarthria, and abnormal mental behaviors (2). Ornidazole, the third generation of novel nitroimidazole derivatives, is widely used, thanks to its fast onset, long half-life, strong antimicrobial activity, and fewer side effects (3, 4). Ornidazole-induced encephalopathy (OIE) is hard to diagnose at its initial stage due to its rarity and secretiveness and may lead to serious complications. Combining with a literature review, we report a case of a delayed diagnosis of encephalopathy induced by prolonged use of ornidazole and summarize the clinical characteristics of OIE to improve the clinical identification of such symptoms.

## Case Report

A 62-year-old man was first admitted to our hospital on October 1, 2020 for several days of unsteady gait, unclear speech, and abnormal mental behaviors. He denied having symptoms of weakness, dizziness, nausea, vomiting, and coughing when drinking water, nor did he have any family history of neurological disease, central nervous system dysfunction, or genetic disorders. He also denied chronic alcohol abuse, gastrointestinal surgical procedures, recurrent vomiting, chronic diarrhea, or nutritional imbalance. He had suffered from hypertension for several years without any other diseases.

On admission, the patient was irritable and easily agitated. His height was 1.75 m and weight was 75 kg. His body temperature was 36.6°C, pulse rate 80/min, respiratory rate 18/min, and blood pressure 145/78 mmHg. Neurological examination showed that he had cerebellar signs such as unsteady gait, dysarthria, and nystagmus. He failed to accurately perform the finger–nose and heel–knee–tibia tests. His Romberg sign was positive and the sensation in the upper and lower limbs decreased slightly. Other neurological and medical examinations showed no abnormalities.

**Figure 1:**
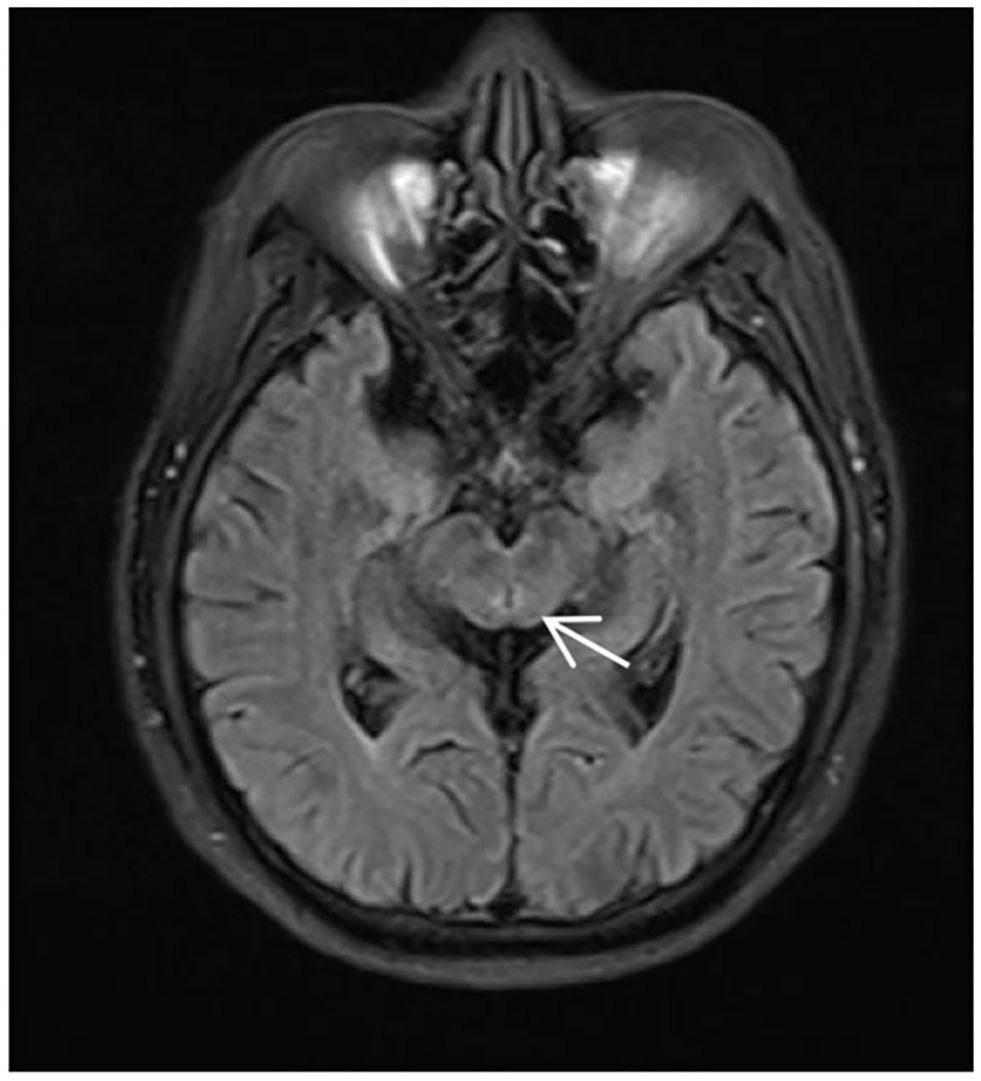
MRI of the patient during his first time in the hospital, T2/fluid-attenuated inversion recovery (FLAIR) showing abnormally high signals around the midbrain aqueduct (*arrow*).

The magnetic resonance imaging (MRI) results only showed mild abnormal signals around the midbrain aqueduct in T2/fluid-attenuated inversion recovery (FLAIR) (Figure 1). Needle electromyographic (EMG) examination was normal. There was no abnormality in the motor and sensory nerve conduction velocity. The compound muscle action potential (CMAP) amplitude of motor nerves was normal, but the sensory nerve action potential (SNAP) amplitude of the sensory nerves decreased in the upper and lower limbs, which indicated damage to the sensory nerves. Laboratory investigations revealed normal levels of routine blood, liver and kidney function, blood glucose, blood ammonia, serum electrolytes, blood clotting function, serum creatine phosphokinase, thyroid function, rheumatologic antibodies, tumor biomarkers, folate, vitamins B1 and B12, and homocysteine.

**Figure 2:**
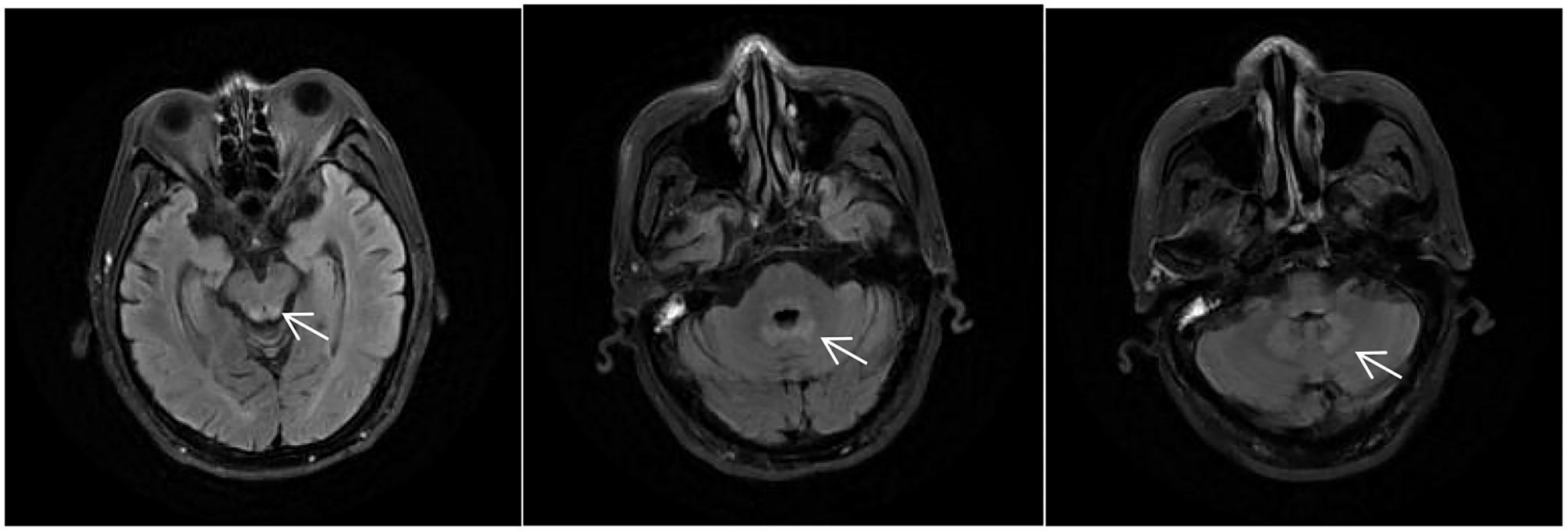
MRI of the patient during his second time in the hospital, T2/fluid-attenuated inversion recovery (FLAIR) showing abnormally high signals around the midbrain aqueduct, around the fourth ventricle, and in the dentate nuclei of the cerebellum bilaterally (*arrows*).

After 3 days of general supportive treatment and administration of vitamins B1 (25 mg, p.o. t.i.d.) and B12 (0.5 mg, p.o. t.i.d.), all of his symptoms almost completely disappeared. He was then discharged from our hospital with unclear diagnosis. On January 26, 2021, he was once again hospitalized, exhibiting much worse symptoms. Neurological examination results were the same as before. A second MRI examination showed abnormal signals around the midbrain aqueduct, around the fourth ventricle, and in the dentate nuclei of the cerebellum bilaterally in T2/FLAIR (Figure 2). Cervical, thoracic, and lumbar MRI examination results were normal. Other negative results included routine cerebrospinal fluid, biochemistry, and etiology, serum anti-intrinsic factor antibodies, and anti-parietal cell antibodies. The serum and cerebrospinal fluid tests were also negative, including central nerve demyelinating antibodies (AQP4, MOG, GFAP, and MBP), peripheral neuropathy antibodies (GM1, GM2, GM3, GM4, GD1a, GD1b, GT1a, GT1b, GQ1b, and sulfatide), paraneoplastic antibodies (CV2, Ri, Yo, Hu, GAD 65, SOX1, and Titin), and oligoclonal bands.

**Figure 3:**
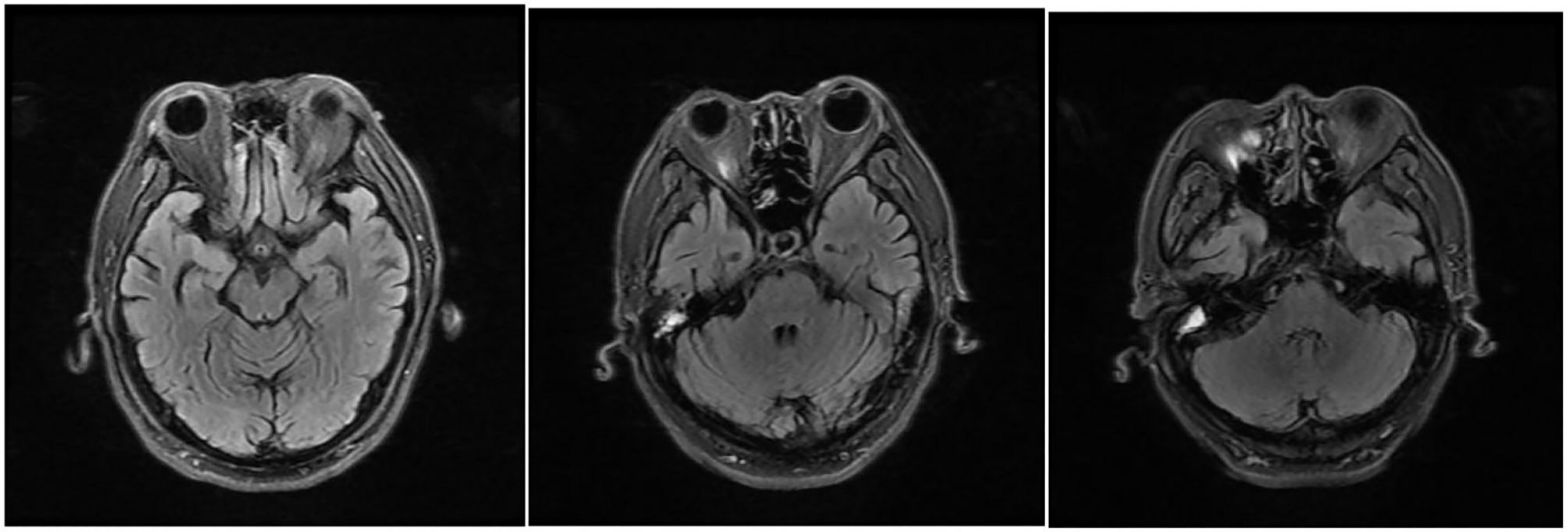
MRI reexamination of the patient after drug withdrawal during his second time in the hospital, T2/fluid-attenuated inversion recovery (FLAIR) showing abnormally high signals around the midbrain aqueduct, around the fourth ventricle, and in the dentate nuclei of the cerebellum bilaterally, which disappeared 9 days after drug withdrawal.

This time, the patient reported a medical history of taking ornidazole (1,000 mg/day) since the end of 2016 to treat proctitis and diarrhea. To prevent the recurrence of the latter disease, he kept taking this medicine even after the symptoms were relieved. During his first time in the hospital, he stopped taking the drug because he failed to bring it, and he did not disclose his medication history of taking ornidazole to the doctor. He resumed taking it after being discharged from our hospital. After the second admission, he was stopped from taking ornidazole and was treated with vitamins B1 (0.1 g, q.d. i.m.) and B12 (0.5 mg, q.d. i.m.). Four days later, his symptoms were significantly alleviated. After 9 days, MRI showed the absence of intracranial lesions (Figure 3) and the main symptoms completely disappeared, except alleviated irritability.

## Discussion

Here, we report the case of a 62-year-old man presenting with cerebellar ataxia and mental symptoms. The man had taken ornidazole for over 4 years, and his symptoms improved after withdrawal of the medicine. After resumption, however, the symptoms recurred. Magnetic resonance imaging showed reversible abnormal lesions that could explain his symptoms, and laboratory tests ruled out other causes. On this basis, he was clinically diagnosed with OIE and was subsequently fully informed of the clear diagnosis and he agreed to the treatment.

The world's first case of OIE was reported from Turkey in 2010, and another three cases from India. All four previously reported patients were females, while this is the first report of a male patient. Below is a summary of the clinical characteristics of the five patients (including our case; see Table 1). The patients were between 23 and 62 years old. The duration of OIE ranged from 35 days to 4 years. Cerebellar ataxia was the common symptom of all five patients, and in three of them, this was accompanied by mental behavior abnormalities such as agitation and irritability. All of them had abnormal lesions in the dentate nucleus of the cerebellum bilaterally, among whom four also had lesions in the corpus callosum and three around the periaqueduct of the midbrain. After discontinuation of ornidazole, the symptoms of all patients improved or vanished, and the lesions of three patients disappeared or were mitigated in the MRI reexamination results. All patients had good prognosis without obvious sequelae.

**Table 1:** Literature review on ornidazole-induced encephalopathy (OIE).

**Authors**	**Sex**	**Age**	**Duration of medication**	**Daily dosage**	**Clinical manifestations**	**Location of MRI lesions**	**Time for symptom improvement after drug withdrawal**	**Follow-up MRI**	**Prognosis**
Taskapilioglu et al. (5)	Female	23	35 days	1,000 mg/day	Ataxia Dysarthria Emotionally labile	Dentate nucleus Corpus callosum	2 days	Lesions disappeared	Good
Gopinath et al. (6)	Female	61	365 days	500 mg/day	Ataxia Dysarthria	Dentate nucleus Corpus callosum Midbrain aqueduct	14 days	Not mentioned	Good
Sekhar K et al. (7)	Female	51	365 days	Not mentioned	Ataxia Dysarthria	Dentate nucleus Corpus callosum Pons dorsal	Not mentioned	Not mentioned	Good
Chouksey et al. (8)	Female	34	3–4 years	Not mentioned	Ataxia Dysarthria Mental disorder	Dentate nucleus Corpus callosum Midbrain aqueduct	60 days	Lesions disappeared	Good
The case reported here	Male	62	4 years	1,000 mg/day	Ataxia Dysarthria Mental disorder	Dentate nucleus Midbrain aqueduct Around the fourth ventricle	4 days	Lesions disappeared	Good

Ornidazole is chiefly metabolized in the liver, and most of it is excreted in the urine as metabolites, with <4% being prototype drugs. *In vivo*, it mainly acts on the DNA of anaerobic bacteria and protozoa, such as amoeba, giardia, and trichomonas, *via* cytotoxic original drugs and intermediate metabolic active products. These drugs and products kill anaerobic bacteria and protozoa by breaking the helical structure or preventing the transcription or replication of their DNA, thus achieving antibacterial and antiprotozoal purposes. Ornidazole is easily absorbed through the gastrointestinal tract, and its plasma elimination half-life is 10.8 ± 1.4 h (9). Ornidazole is widely distributed in human tissues and body fluids and can easily penetrate the blood–brain barrier with high lipid solubility. In most tissues, including the central nervous system, the concentration of ornidazole can reach 60–100% that of the plasma (10). As a result, it will cause adverse neurological reactions. Compared with MIE, however, OIE is exceptionally rare, and only a few cases have been reported worldwide. Sørensen et al. (11) retrospectively analyzed the clinical data of 136 MIE patients averaging 56.8 years old. These patients took metronidazole against gastrointestinal infection or for other reasons. The duration of taking metronidazole ranged from 2 days to 8 years, with an average of 101.6 days, a lower quartile of 19.5 days, a median of 35 days, and an upper quartile of 63 days. The duration of treatment was longer than 1 year for 3.9% of patients. The cumulative dosage ranged from 5 to 2,000 g, with an average of 125.7 g, a lower quartile of 36 g, a median of 65.4 g, and an upper quartile of 110.8 g. The average daily dosage was about 1.24 g. The average duration of treatment before the onset of the first symptoms from the central nervous system was 47.2 days. Our case had taken ornidazole for nearly 4 years before developing neurological symptoms. Patients with OIE were reported to have taken the drug for at least 35 days before symptom onset, with a cumulative dosage of 35 g. Because only a few cases of OIE have been reported, a statistical comparison with MIE in terms of duration and dosage for treatment is quite challenging. Ornidazole can be converted into levornidazole *in vivo*. Levornidazole shows similar clinical therapeutic effects to, and fewer adverse reactions than those of metronidazole in the central nervous system (12). These might be the reasons for the low incidence of OIE.

Sørensen et al. (11) retrospectively analyzed the clinical manifestations of patients with MIE and found that dysarthria was the most common, followed by unsteady gait, limb disharmony, mental state change, multiple peripheral neuropathies, and eye movement disorders. The clinical manifestations of OIE resemble those of MIE, mainly cerebellar ataxia such as slurred speech, unsteady gait, and uncoordinated limb movements. In some patients, the symptoms are also accompanied by emotional agitation, irritability, and abnormal mental behaviors such as gibberish, and a few have peripheral nerve damage such as limb numbness. Magnetic resonance imaging plays an important role in diagnosing diseases. T2-weighted imaging (T2WI) and FLAIR sequences often show abnormally high intracranial signals (13). In MIE, lesions in the dentate nucleus of the cerebellum bilaterally are the most common, accounting for 90%. Fewer than that are the lesions in the corpus callosum mostly found in the splenium, accounting for 44%. Other lesions are in the brainstem, such as the midbrain, pons, and medulla oblongata, while a few lesions are located in the basal ganglia and white matter (11). The distribution of OIE is similar to that of MIE. All the reported OIE cases had cerebellar dentate nucleus lesions, which can be reversed after drug withdrawal.

The pathogenesis of OIE is unclear at present. The mechanisms of metronidazole and ornidazole are similar as they both belong to nitroimidazole drugs. Both MIE and OIE belong to the type 3 antibiotic-associated encephalopathy (AAE) (14). Since the MRI result of nitroimidazole-induced encephalopathy (NIE) is similar to that of non-alcoholic Wernicke encephalopathy (WE), most patients with NIE have gastrointestinal diseases and abnormal liver function, which may lead to vitamin B1 deficiency or malabsorption in the body (15). The metabolites of nitroimidazole drugs may be converted into analogs of thiamine and may be antagonistic to vitamin B1 in the body, also resulting in vitamin B1 malabsorption (16). Some scholars, therefore, believe that there may be an overlapping pathophysiological mechanism between NIE and WE. Nevertheless, other lesions with the characteristics of WE, such as medial thalamic lesions and papillary body lesions, are rarely found in NIE, indicating differences between the two. Ornidazole-induced encephalopathy can lead to abnormal mental behaviors. Some scholars hold that a possible reason is that nitroimidazole drugs can inhibit monoamine oxidase, thereby reducing the decomposition of dopamine and causing accumulative dopamine in the body, further resulting in mental disorders (17).

The main differential diagnoses of OIE include WE, toxic encephalopathy, metabolic encephalopathy, and demyelinating disease of the central nervous system. The patient denied having a history of chronic alcohol abuse, gastrointestinal surgical procedures, recurrent vomiting, chronic diarrhea, or nutritional imbalance that may cause WE, and the serum vitamin B1 level was normal and the serum anti-intrinsic factor antibodies and anti-parietal cell antibodies were negative. None of these support the diagnosis of WE. Demyelinating diseases of the nervous system such as neuromyelitis optica (NMO) and myelin oligodendrocyte glycoprotein (MOG)-associated ones should be considered, but they can be excluded based on the negative results of aquaporin 4 (AQP4), MOG, and spinal MRI. Sensory nerve damage in the upper and lower limbs can be explained by the toxicity of ornidazole, which is the main adverse reaction of the nervous system induced by nitroimidazoles (1).

In this case, OIE was not difficult to identify according to the clinical manifestations, previous medication history, imaging features, and outcomes after drug withdrawal. Like other previously reported cases, the symptoms and intracranial lesions were abated rapidly after the timely withdrawal of medication. This patient was treated with vitamins B1 and B12. Therefore, this also might have contributed to his recovery. The general prognosis for him was good without obvious neurological sequelae.

## Conclusion

Ornidazole is widely used in clinical practice, yet the encephalopathy induced by ornidazole is easily missed and misdiagnosed because it is rather rare. For correct diagnosis, a clear drug use history can be of help. In terms of treatment, the lesions in the cerebellum, corpus callosum, and brainstem, mainly manifested by cerebellar ataxia, dysarthria, and mental symptoms, are reversible. After drug withdrawal, these clinical symptoms recover quickly, with good prognosis. For clear pathogenesis, however, further investigations are required.

## Data Availability Statement

The original contributions generated for the study are included in the article/supplementary material, further inquiries can be directed to the corresponding authors.

## Ethics Statement

Written informed consent was obtained from the individual(s) for the publication of any potentially identifiable images or data included in this article.

## Author Contributions

XL designed the case report. RT wrote the article. JL, YL, and TW collected data's. XL, YZ, and YM revised the manuscript. All authors contributed to the manuscript revision and approved the final manuscript.

## Funding

This study was supported by the National Natural Science Foundation of China (grant no. 81971204).

## Conflict of Interest

The authors declare that the research was conducted in the absence of any commercial or financial relationships that could be construed as a potential conflict of interest.

## Publisher's Note

All claims expressed in this article are solely those of the authors and do not necessarily represent those of their affiliated organizations, or those of the publisher, the editors and the reviewers. Any product that may be evaluated in this article, or claim that may be made by its manufacturer, is not guaranteed or endorsed by the publisher.
